# A finite element analysis of novel vented dental abutment geometries for cement‐retained crown restorations

**DOI:** 10.1002/cre2.33

**Published:** 2016-06-14

**Authors:** Lucas C. Rodriguez, Juliana N. Saba, Clark A. Meyer, Kwok‐Hung Chung, Chandur Wadhwani, Danieli C. Rodrigues

**Affiliations:** ^1^ Department of Bioengineering University of Texas at Dallas Richardson Texas USA; ^2^ Department of Restorative Dentistry University of Washington Seattle Washington USA

**Keywords:** Abutment, cement, dental, peri‐implant disease, peri‐implantitis, venting

## Abstract

Recent literature indicates that the long‐term success of dental implants is, in part, attributed to how dental crowns are attached to their associated implants. The commonly utilized method for crown attachment – cementation, has been criticized because of recent links between residual cement and peri‐implant disease. Residual cement extrusion from crown‐abutment margins post‐crown seating is a growing concern. This study aimed at (1) identifying key abutment features, which would improve dental cement flow characteristics, and (2) understanding how these features would impact the mechanical stability of the abutment under functional loads. Computational fluid dynamic modeling was used to evaluate cement flow in novel abutment geometries. These models were then evaluated using 3D‐printed surrogate models. Finite element analysis also provided an understanding of how the mechanical stability of these abutments was altered after key features were incorporated into the geometry. The findings demonstrated that the key features involved in improved venting of the abutment during crown seating were (1) addition of vents, (2) diameter of the vents, (3) location of the vents, (4) addition of a plastic screw insert, and (5) thickness of the abutment wall. This study culminated in a novel design for a vented abutment consisting of 8 vents located radially around the abutment neck‐margin plus a plastic insert to guide the cement during seating and provide retrievability to the abutment system.Venting of the dental abutment has been shown to decrease the risk of undetected residual dental cement post‐cement‐retained crown seating. This article will utilize a finite element analysis approach toward optimizing dental abutment designs for improved dental cement venting. Features investigated include (1) addition of vents, (2) diameter of vents, (3) location of vents, (4) addition of plastic screw insert, and (5) thickness of abutment wall.

## Introduction

Dental implants have been used to improve the quality of life of millions of patients over the past 40 years or so (Hamdan et al. 2013; Menassa et al. 2016; Packer et al. 2009; Scully et al. 2007; Strassburger et al. 2006; Vieira et al. 2014). Recently, however, systematic reviews in the dental literature are indicating one of the factors associated with long‐term success relates to how the implant‐crown is attached (Chaar et al. 2011; Ma and Fenton 2015; Wismeijer et al. 2014; Wittneben et al. 2014). One of the most commonly used crowns to implant attachment methods is cementation to an abutment. Although the processes of using cement for crown retention are well established and something dentists have been performing on natural teeth for 100 years, they present challenges where implant restoration is concerned. One major issue with implant restoration is the issue of residual cement extrusion at the crown‐abutment interface or lute margin site. A link between peri‐implant disease and residual cement has been established in the literature, with excess cement associated with signs of peri‐implant disease in approximately 80% of cases (Wilson 2009).

Cement‐retained restorations are often preferred over screw‐retained prostheses because of their simplicity, improved esthetics, control of occlusion, economics, and passivity of fit (American Academy of Periodontology 2013; Wilson et al. 2014). However, during the process of cementing the restoration onto an implant abutment, cement extrusion can occur. The excess cement extrusion is a result of several factors including force used in seating, amount of cement used, cement composition, site of application, clinical skill, and implant abutment geometry (Wadhwani et al. 2015). Peri‐implant disease is an inflammatory reaction affecting the soft and hard tissues surrounding an implant (Wilson et al. 2014). Residual cement extrusion into the peri‐implant tissues has been associated with this disease resulting in a variety of complications including soft tissue inflammation, foreign body giant cell reaction, soreness, suppuration, bleeding on probing, and loss of the implant supporting bone (Patel et al. 2009; Wadhwani et al. 2011a; Wilson et al. 2014). Other exacerbating factors have also been cited. Linkevicius et al. (2013), in a retrospective case analysis, reported implants with remnants of cement in patients with history of periodontitis may be more likely to develop peri‐implantitis compared with patients without history of periodontal disease. Although the exact etiology of peri‐implant disease is not fully understood, there appears to be some commonality with periodontal disease in that both have similar microbial profiles. Apart from a proposed microbial etiology, other factors may play a role in triggering residual cement related peri‐implant disease (Rodrigues et al. 2013; Wadhwani et al. 2014). It has also been demonstrated that the adequate removal of excess cement can result in resolution of the peri‐implant disease if addressed early (Scully et al. 2007). Thus, removal and prevention of all residual cement should be regarded as a high priority during implant restorative procedures (Korsch et al. 2013; Linkevicius et al. 2013; Ramer et al. 2014; Squier et al. 2001; Wadhwani et al. 2011a; Wadhwani et al. 2014; Wilson et al. 2014).

Regarding cement use, it has been recognized that overfilling a crown with cement can result in higher probability of cement extrusion onto the soft tissues and implant surfaces (Wadhwani and Pineyro 2012). Conversely, under filling the crown can result in inadequate crown retention and failure. Crown and abutment designs play a crucial role in how the luting cement works and flows. Ideally the abutment‐crown configuration should allow for an optimum layer of luting cement; both providing adequate crown retention yet limiting excessive flow. Abutment designs have been reported, which can control cement flow. Examples of crown and abutment designs specifically to direct cement flow include venting and internal inserts (Patel et al. 2009; Schwedhelm et al. 2003; Wadhwani and Chung 2014; Wadhwani et al. 2011b; Wadhwani et al. 2013).

The purposes of this study were to (1) identify key abutment features, which would improve dental cement flow characteristics, and (2) understand how these features would impact the mechanical stability of the abutment under functional loads. To this end, several implant abutment designs have been developed, dedicated to (1) provide flow channels for dental cement within the crown‐abutment system to provide laminar flow, (2) increase volume within the abutment to decrease the risk of residual cement extrusion (decrease risk of overfilling crown), (3) increase surface area in contact with cement, thereby increasing retentive strength of the crown, and (4) add a screw insert to guide cement flow and protect the screw head. This information has the potential to direct future abutment design in an effort to improve flow of dental cements and potentially reduce the risk of residual cement extrusion by allowing for a standardization of cement loading volumes prior to seating.

## Materials and Methods

The methodology used for this study consisted of virtual modeling using specialized computer software capable of determining fluid dynamic flow. Evaluation was further performed by using surrogate models of dental abutments. Finite element analysis (FEA) was undertaken to determine the number, location, and diameter of vents that could be placed within the abutment without substantive structural yield/failure under load.

### Abutment design

Traditionally, implant abutment designs have been based on tooth form preparations resembling a frustum (truncated cone) (Wadhwani et al. 2011b). The center of the abutment typically has a hollow screw channel that is commonly occluded with Teflon tape (Tarica et al. 2010). The concept of utilizing the screw access channel as well as altering the configuration of the abutment was the basis of the experimental question.

Abutments in this study were designed to fit two major criteria: (1) provide increased cement flow throughout the entire geometry, and (2) increase the cement capacity (volume) within the crown‐abutment system.

**Figure 1:**
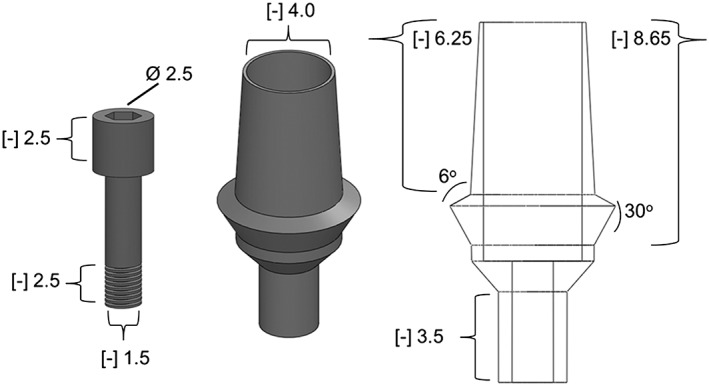
Abutment design features.

Abutment and screw design features were adapted from the Semodos implant system (Bego, Bremen, Germany) (Tang et al. 2012). The base model features a tapered, conical (6^o^ wall taper, 30^o^ wing taper) abutment (opening Ø 4 mm × 6.25 mm) with a connection depth of 3.5 mm and a cement channel of 8.65 mm (Fig. 1).

**Figure 2:**
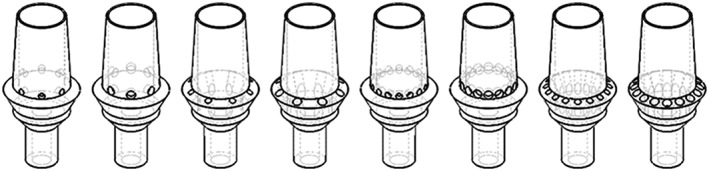
Adaptations of the base abutment to include vents of various diameter and location.

The abutments were subsequently modified to allow for venting of the system (Fig. 2). Here, vents were incorporated into the geometry in two locations (abutment neck and abutment margin) and two sizes (0.5 mm diameter and 0.7 mm diameter).

### Plastic insert flow analysis

**Figure 3:**
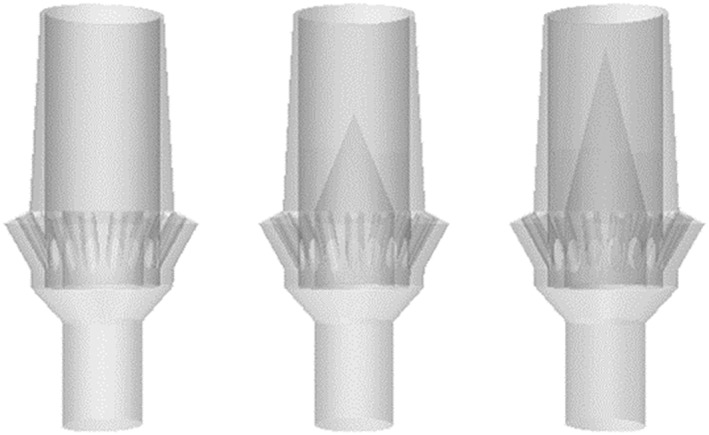
Translucent abutments illustrating (left) no plastic insert, (middle) a short plastic insert, and (right) a tall plastic insert.

**Figure 4:**
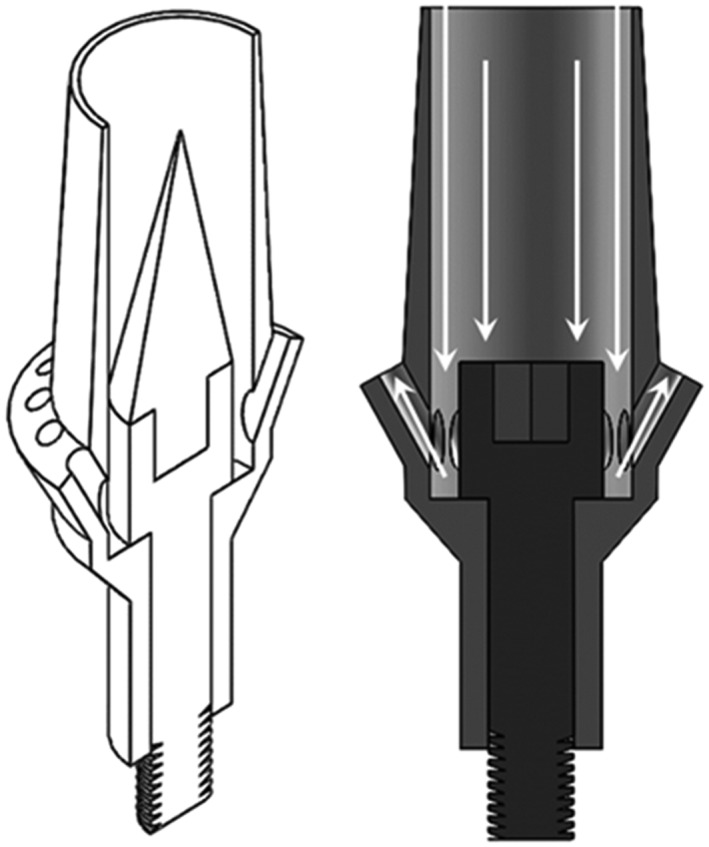
Proposed flow directions of abutments with plastic insert.

By utilizing a plastic insert into the screw head within the abutment screw channel prior to cementation, the screw head remains protected from the cement and can still be retrieved. Parameters of interest to this phase of the study included the height and taper of the insert to optimize the flow characteristics of fluid in the geometry (Figs. 3, 4; Table 1). star‐ccm+ computational fluid dynamics (CFD) software (CD‐Adapco, Melville, NY) was used to determine the most optimal fluid dynamics with regard to minimizing dead space (unfilled space) within the abutment. Dead space was the primary parameter of interest here because of the assumption that reducing dead space in the abutment would reduce the ability for cement (during seating) to create air pockets. These systems are not designed to assume air pocket or void formation. These voids (areas without cement) will result in a discrepancy between the amount of cement loaded into the intaglio of the crown and the amount of cement, which the abutment can accommodate. Models were initially built in solidworks (computer aided design (CAD) software; Dassault Systemes, Waltham, MA) and transferred to the CFD software as stereolithography (.stl) files. Abutments were meshed using a polyhedral mesh with proximity refinement (minimum cell size of 0.05 mm and a maximum cell size of 2.5 mm).

**Table 1:** Height and taper angle of inserts investigated in insert flow analysis.

	Height (mm)	Taper angle
Insert 1	1.04	39.38
Insert 2	2.08	58.70
Insert 3	3.13	67.93
Insert 4	4.17	73.09
Insert 5	5.21	76.32
Insert 6	6.25	78.54

For the computations, the coronal portion of the screw access channel of the abutment was defined as a fluid inlet with fluid flow of 5 mm/s. The abutment was further modified with up to 16 vents located within the margin of the abutment (defined as fluid outlets). The cement simulations were run using viscosity properties acquired using dynamic rheometry (Discovery HR‐3 Hybrid Rheometer, TA Instruments, New Castle, DE) (250 Pa/s), which closely resembles that of freshly mixed TempoCem – a zinc oxide/eugenol cement (DMG, Dental Milestones Guaranteed). A steady state solver (time independent) was used and was run until residuals converged below 1 e^‐4^ evidencing the simulation was complete. Results were represented using fluid velocity to determine the rate of fluid flow within the abutment system during seating (mm/s).

As a means of initially validating the optimum insert after the CFD analysis was completed, models (crown and abutment with insert) were fabricated by 3D‐printing after scaling 5× (3D‐printed test models were five times the size of abutment design) using a Connex350 (Stratasys, Eden Prairie, MN). Printed models (*n* = 3) with insert dimensions (angle and length; Table 1) of the optimal flow characteristics post‐CFD investigation were produced. Each abutment had a crown form fabricated to be seated onto the abutment with a crown‐abutment cement lute space of 50 µm provided. The printed crown was loaded with a zinc oxide eugenol dental cement (Tempocem, DMG) and subsequently seated onto the printed abutment with plastic insert using a mechanical testing system (MTS Bionix, Model 370; MTS Systems Corporation, Eden Prairie, MN) at 0.5 mm/s (Wadhwani et al. 2014). After the dental cement was completely set, the abutment was sectioned both through the sagittal and transverse planes to allow for visualization into the cemented system. The goal of this particular analysis was to ensure that cement filled the available space within the abutment without gaps or holes.

### Finite element analysis (channel depth, wall thickness, vent number, vent diameter, and vent location)

Finite element analysis was conducted to determine the impact adding venting features had on the abutments mechanical stability. Channel depth, number of vents, vent diameter, vent location, and wall thickness were all examined. FEA evaluated the resulting maximum von Mises stresses of each design, location of the maximum stresses, and stress risers within each geometry investigated in relation to the yield stress values of commercially pure titanium.

#### Three‐dimensional finite element modeling

The three‐dimensional CAD geometry models designed in solidworks (Dassault Systemes) were transferred using standard ACIS text files into abaqus fe (Dassault Systemes) to generate meshes and perform the numerical simulation. All components were meshed with tetrahedral elements of type C3D10 type elements readily available in abaqus element library. Meshes were generated varying element size parameters to verify mesh independence for each geometry. All results had less than 2.6% variation between mesh sizes.

#### Boundary conditions and constraints

In this study, we assumed the abutments were homogeneous, linear elastic, and had isotropic mechanical properties. Material properties for abutment components (commercially pure titanium grade 4) were collected from reliable resource and published data (RMI Titanium Company 2000). The models were constrained in *X*‐, *Y*‐, and *Z*‐directions.

#### Loading conditions

A distributed force of 250 N (average maximum human bite force) was applied onto the top surface of the abutment in the form of pressure (Allum et al. 2008; Biswas et al. 2013; Cornell et al. 2009; Quaresma et al. 2008; Takaki et al. 2014; Tang et al. 2012). The area of the contact surface was 1.70 mm^2^ for all abutments tested resulting in a pressure of 141.24 N/mm^2^. All pressures were loaded normal to the contact surface (loaded parallel to the long axis of the abutment). Subsequently, a concentrated force (170 N total) was loaded 30^o^ offset from the abutment surface to investigate the impact of non‐normal loading in abutments with various abutment wall thicknesses (0.15 mm vs. 0.65 mm).

#### Finite element analysis

In this study, we selected nine novel implant models to investigate the stress distribution in the abutment systems. Parameters of interest included channel depth, wall thickness, vent number, vent diameter, and vent location. For a direct and systematic comparison, the same load conditions, boundary conditions and constraints were applied in all three models. abaqus/Standard solver (installed to a desktop computer with a 3.20 GHz processor and 8 GB memory and ran under Windows 8 Enterprise operating system) was used to analyze model data and perform the stress analysis in the abutments subject to loading.

## Results

### Plastic insert flow analysis

**Figure 5:**
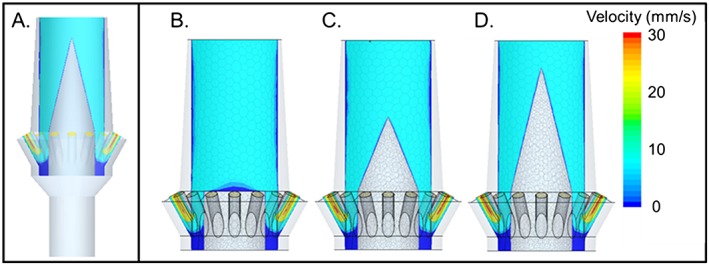
Results of the computational fluid dynamics analysis on plastic insert impact on flow. (A) illustrates the symmetry plane used to represent the data, (B) represents the flow without a plastic insert, (C) represents an insert with 3.13‐mm height and 70^o^ taper angle, and (D) represents the optimal insert investigated by this stage (5.21‐mm height and 75^o^ taper).

Results from the CFD modeling are illustrated in Figure 5. Figure 5A shows the symmetry plane used to visualize results. Figure 5B–D illustrates the trend which was noted during the simulations, increasing the insert height and taper improved flow characteristics within the system. Figure 5B represents a model without a plastic insert. Figure 5C represents an insert with 3.13‐mm height and 70^o^ taper, and Figure 5D represents the insert with 5.21‐mm height and 75^o^ taper. The color scale is representative of the fluid velocity in these locations, which gives an idea of areas of low fluid flow, which can result in air in the system (blue). The 5.21 mm × 75^o^ taper demonstrated the most aerodynamic characteristics of the inserts examined and was selected to be utilized in the subsequent 3D‐printed evaluation experiment.

**Figure 6:**
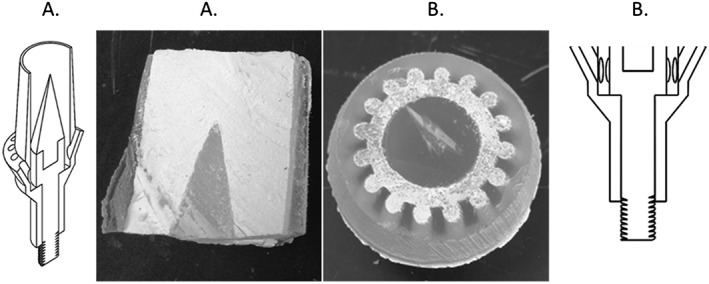
Results of fluid flow evaluation experiment. The abutment and insert were printed with semi‐translucent plastic to allow for visualization (A and B). (A) Represents the sagittal plane, and (B) represents the transverse plane.

Figure 6 shows the results of the 3D‐printed evaluation experiment using a 3D‐printed crown and abutment with the plastic insert optimized in the CFD study previously. Figure 6A represents the sagittal plane, and Figure 6B represents the transverse plane, which were cut and examined. As illustrated, the cement was able to flow throughout the entire geometry, and each of the 16 vents was completely filled, demonstrating completely uniform fluid flow throughout the entire system (Fig. 6B).

**Figure 7:**
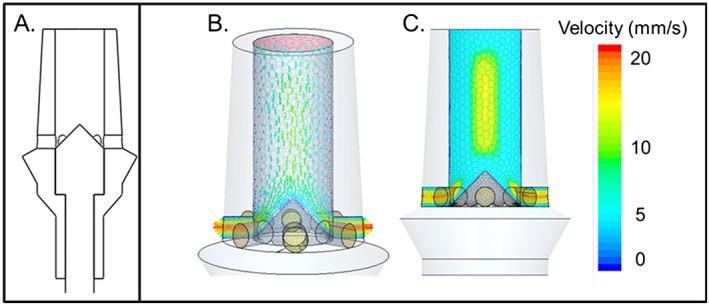
Computational fluid dynamics analysis of amended abutment geometry to reduce cement flow “dead space” within the system. (A) Represents the line drawing of the abutment cut through the sagittal plane, (B) represents the vector image of the vent flow through the abutment, and (C) represents the filled flow pattern of a 45^o^ insert to enable laminar flow through the abutment with no “dead space” in relation to Figure 5.

Figure 7 illustrates the results of increasing the abutment wall thickness from 0.15 mm (previous) to 0.65 mm in an effort to remove the “dead space” represented as blue in the posterior portion of each of the previously invested abutment geometries (Fig. 5). By increasing the thickness of the abutment wall, the abutment came into contact with the screw head and insert and prevented fluid flow into the gap (Fig. 7).

### Finite element analysis (channel depth, wall thickness, vent number, vent diameter, and vent location)

**Figure 8:**
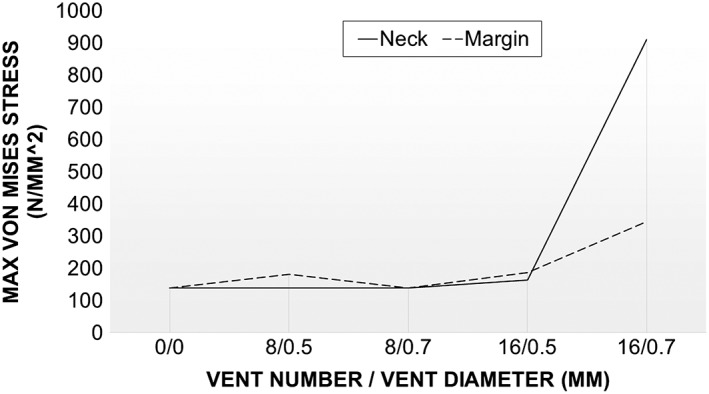
Maximum stress (N/mm^2^) of vented dental abutments with reference to vent location, vent number, and vent diameter.

**Figure 9:**
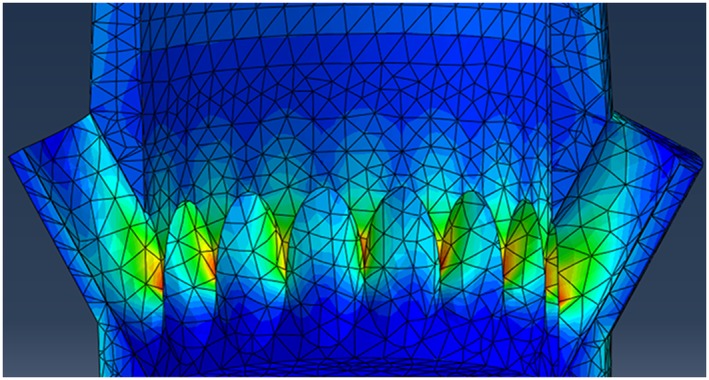
Sagittal view illustrating stress risers due to vent diameter being too large in the 16 vent 0.7‐mm vent diameter models.

Further investigation of the key parameters (vent number, diameter, and location) was conducted on each of the abutments outlined in Figure 2. Results of this study were summarized in Figure 8. As illustrated, the abutment geometries were all mechanically similar to the control group (0 vents) until the 16 vent with 0.7‐mm vent diameter abutment. At this point, the distance between the vents was small enough to cause significant stress risers (Fig. 9) (margin and neck).

**Figure 10:**
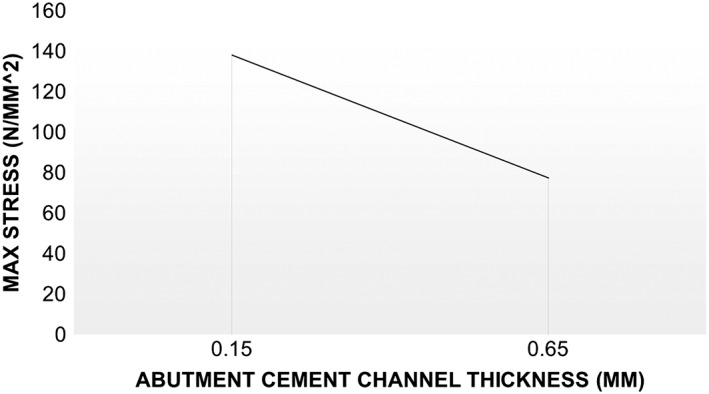
Effect of abutment wall thickness of 8 vented neck abutments.

The investigation of the impact of increasing abutment wall thickness to match that amended for the plastic insert study (from 0.15 to 0.65 mm, Fig. 7) predictably demonstrated that the increase in abutment wall thickness (with all other features remaining constant) reduced the maximum stresses exhibited on the abutment by 56% (Fig. 10) and by 51% when loaded at 30^o^ offset.

**Figure 11:**
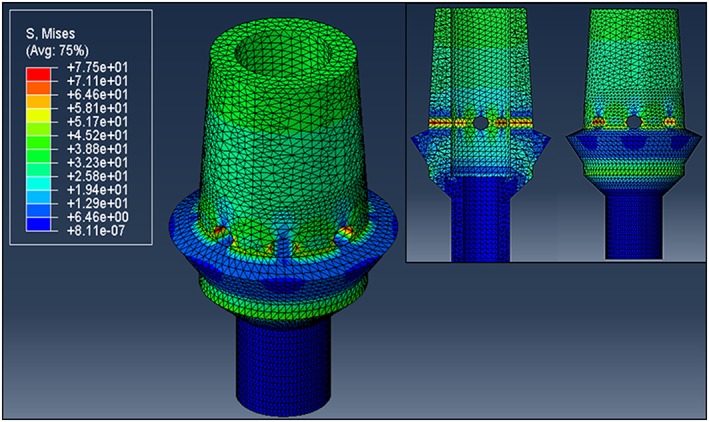
Stress distribution in thick walled (0.65 mm), 8 neck vent (0.7‐mm diameter) abutment. Von Mises units in N/mm^2^.

The stress distribution on the thick‐walled abutment remained uniform through the coronal portion of the abutment with small stress risers on each side (left and right) of the vents in the abutment geometry because of the normal loading of the implant (Fig. 11).

## Discussion

The aim of this study was to understand how abutment features designed to allow for venting within the crown‐abutment system would impact the mechanical stability of the abutment. This understanding could then be utilized in the redesign of the abutment to allow for venting and thus improve the flow of dental cements within the system. Once predictable cement flow is achieved, the precise volume of cement preloaded into the crown for the abutment‐crown seating procedure can be standardized. This standardization of cement volume in the abutment‐crown seating should reduce the risk of unwanted, excess dental luting cement leakage. The overall goal of this study was to utilize finite element analysis tools to preliminarily investigate new abutment designs as a means of yielding evidence for future in vitro bench top models and clinical investigations.

This study was conducted in two primary phases: (1) design of novel abutment geometries to improve cement flow through the crown‐abutment system, and (2) test these abutment geometries to evaluate mechanical stability using finite element analysis tools. CAD tools have made the rapid redesign of implantable devices much more accessible and cost effective. FEA has been the most influential tool available in the simulation of dental restorations under various loading scenarios (Wakabayashi et al. 2008). These simulations provide in‐depth information about the virtual materials, which yield guidelines for iterative designs. Further, the precision with which these tools can predict mechanical stability and yield/failure mechanisms is helpful in decreasing the incidence of redundant benchtop and clinical experimentation.

The new abutment designs took several factors into consideration. Because the goal of the novel abutment design was to allow for complete laminar flow of cement through the system, the vents needed to be situated toward the terminal portions of the geometry (the neck or the margin). The optimal way to allow for such venting was to utilize the interior portion of the abutment body. Thus, the cement channel was developed to allow cement to flow into and through the entire abutment body. The concern with allowing cement to flow through the abutment body in existing abutment geometries stemmed from the need to have the abutment system being retrievable. The clinician must be able to gain access to the screw retaining the abutment to the implant in order to remove the abutment if necessary. To allow for the continued retrievability of the abutment system, the screw insert (Figs. 3, 4, 5, 6) was developed to protect the screw from cement flowing into the abutment body.

Wadhwani et al. (2012) demonstrated that cement application in luting implant‐supported crowns varies widely. There is no standard for how cement should be applied for the best results in reference to crown retention and lack of cement extrusion. This discrepancy in application techniques is most noticeable in the amount/volume of cement ultimately loaded in the system. If the lute space between the crown and the abutment was 50 µm, and Teflon tape was used to plug the screw channel, only ~5 mm^3^ of dental cement would fill the system before extrusion would occur. However, the crown, grossly filled, could typically hold over 100 mm^3^. The vented geometries designed in this study (Fig. 2) could hold up to 70 mm^3^; increasing the volume of cement, which could be occupied by the system by 1300%, reducing the risk of overfilling the crown, and resulting in a large volume of residual cement.

It was hypothesized that when vents were utilized in the abutment, the dental cement could continue to flow throughout the abutment during the entire crown seating. These vented abutments would allow the dental cement to completely fill the open space in the abutment, thus increasing the mechanical stability of the geometry and decreasing the risk of overfilling the system with dental cement. The screw insert was designed to allow for the improvement of fluid dynamic flow while still protecting the screw head from coming into contact with dental cement (to ensure the abutment system could still be retrieved if necessary). Fluid dynamic modeling enabled the systematic evaluation of the different insert heights and taper angles (Fig. 5). These CFD tools made possible the visualization of the cement flow within the abutment geometry, which until recently had not been possible (Wadhwani et al. 2014). Despite the length and angle of the screw insert having an effect on the cement flow through the abutment geometry, the effects are effectively similar. However, each of these simulations indicated a large volume of dead space within the abutment toward the abutments interior margin. Because of the identification of “dead space” where fluid did not flow in the system, the abutment geometry was amended by increasing the abutment wall thickness to prevent flow into the space between the abutment and screw. These results (Fig. 7) illustrated the greatest improvement of fluid dynamic geometry investigated in this study. Additional testing utilizing 3D‐printing technology is currently being conducted to determine if venting at the neck or venting at the margin is more beneficial with regard to residual excess cement. Unpublished preliminary data has suggested that there is no statistical difference resulting from the vent location (neck versus margin).

The finite element analysis phase of the current study was aimed at investigating the impact of flow improvement features (cement channel, screw insert, and vents) would have on the mechanical stability of the abutment. From a mechanical standpoint, the perforation of the abutment neck with vent channels could have led to a decrease the geometry's mechanical stability by increasing stress risers in an already at‐risk location of the abutment. However, from a design perspective, it was hypothesized that vent channels could be incorporated to improve cement flow and then the abutment design could be amended to compensate for such mechanical risks. Mastication is a highly dynamic mechanical process. According to Takaki et al. (2014), the average maximum bite force of men and women independent of their age is 285 and 254 N respectively. While men demonstrated a 12% higher bite force than women, this discrepancy was not statistically significant (*P* > 0.05) (Takaki et al. 2014). This average maximum bite force was used in the design of this FEA in an attempt to investigate the mechanical response of the abutments designed in a worst‐case scenario. As illustrated in Figure 8, under a normal (loaded parallel to the long axis of the abutment) load orientation at 250 N, the abutments investigated were capable of withstanding normal bite forces adequately prior to the 16 vent, 0.7 mm vent diameter abutments. Regardless of vent location (neck or margin), the abutment exhibited a dramatic increase in maximum stress (488% increase in neck and 134% increase in margin) once the vent diameter exceed 0.5 mm while the vent number remained 16. Because of the sharp stress rise with these 16 vent abutments, the 8 vent abutment was selected for investigation of abutment wall thickness impact on mechanical integrity (Figs. 10, 11). Results demonstrated that increasing the channel thickness (0.15–0.65 mm) reduces the maximum stresses on the abutment by 56% when loaded normal to the abutment and by 51% when loaded 30^o^ offset from the abutment. This amended wall thickness, channel diameter, and vent number where found suitable for normal oral environmental forces (e.g., mastication forces) in the finite element analysis. Because the flow analysis confirmed that the thick cement wall resulted in less “dead space” in the abutment system and the FEA results demonstrated a reduction in maximum stress with these abutments, this novel vent design will now be further characterized using in‐depth flow analysis and laboratory benchtop mechanical modeling in future follow‐on studies (Figs. 7, 10, and 11). These abutment designs will be fabricated and tested using standard testing methods to evaluate its potential clinical utility in worst‐case loading scenarios. Preliminary evidence has demonstrated that these designs withstand forces exceeding that of normal mastication forces and meet the requirements necessary for clinical utilization. A follow‐on study will investigate the full mechanical characterization of this design.

Finite element analysis is certainly not without its limitations. One limitation to these computational models is they are based on the assumptions input by the user of the software. These studies require validation. Future studies will validate these results by investigating the novel abutment geometry in a mechanical bench top model to determine static, fatigue, and retentive stresses on the abutment in 3D‐printed laser sintered titanium. Additional studies will also investigate how dental cement rheological properties impact flow within the abutment system as well. The flow behavior of commercially available dental cements needs to be better investigated, as cements will exhibit particular rheological behavior depending on their chemical composition and abutment placement technique. This flow behavior discrepancy (pseudoplasticity) implies that the flow of a particular cement brand may be more susceptible to changes in pressure (shear‐thinning) during crown placement, which may increase its risk of extrusion and accumulation in peri‐implant tissues.

## Conclusion

In conclusion, venting dental abutments can be accomplished to allow for improved dental cement flow within the system. However, the abutment geometry must be carefully designed to ensure mechanical stability. This improved, completely laminar dental cement flow is reliable and reproducible. Knowing how cement will behave in a crown‐abutment system will allow for predictable cementation procedures, which will enable the standardization of cementation techniques for clinicians.

## Conflict of Interest

None declared.
